# A New Antidote for Morphine

**Published:** 1890-06-14

**Authors:** 


### A NEW ANTIDOTE FOR MORPHINE.

Professor Bokai, of Klausenburg, recommends picrotoxin as an antidote in poisoning by morphine. Morphine depresses or paralyses the respiratory centre, and picrotoxin acts as a direct stimulant or excitor of the same. Morphine, too, induces diminution of the blood pressure, while picrotoxin stimulates the vasoconstrictor centre in the medulla. Hitherto the only direct physiological antidote to morphine known has been atropine, but Dr. Bokai considers picrotoxin to be preferable in several respects. He suggests that it might be useful in averting asphyxia from chloroform, though he has not put it to a practical test in this condition.

